# Cooperative DF Protocol for MIMO Systems Using One-Bit ADCs

**DOI:** 10.3390/s22207843

**Published:** 2022-10-15

**Authors:** Tae-Kyoung Kim

**Affiliations:** Department of Electronic Engineering, Gachon University, Seongnam 13120, Korea; tk415kim@gmail.com

**Keywords:** multi-input multi-output, cooperative communication, pairwise error probability, one-bit ADCs

## Abstract

This study considers a detection scheme for cooperative multi-input–multi-output (MIMO) systems using one-bit analog-to-digital converters (ADCs) in a decode-and-forward (DF) relay protocol. The use of one-bit ADCs is a promising technique for reducing the power consumption, which is necessary for supporting future wireless systems comprising a large number of antennas. However, the use of a large number of antennas remains still limited to mobile devices owing to their size. Cooperative communication using a DF relay can resolve this limitation; however, detection errors at the relay make it difficult to employ cooperative communication directly. This difficulty is more severe in a MIMO system using one-bit ADCs due to its nonlinear nature. To efficiently address the difficulty, this paper proposes a detection scheme that mitigates the error propagation effect. The upper bound of the pairwise error probability (PEP) of one-bit ADCs is first derived in a weighted Hamming distance form. Then, using the derived PEP, the proposed detection for the DF relay protocol is derived as a single weighted Hamming distance. Finally, the complexity of the proposed detection is analyzed in terms of real multiplications. The simulation results show that the proposed detection method efficiently mitigates the error propagation effect but has a relatively low level of complexity when compared to conventional detection methods.

## 1. Introduction

A multi-input–multi-output (MIMO) system is a simple but powerful solution for improving system performance by using multiple antennas [1,2,3,4,5]. To fully exploit its advantages, most standard groups have adopted multiple antennas for future wireless standards. Recently, the use of a large number of antennas has attracted interest in spectral/power-efficient method massive MIMO systems. However, the increase in the number of antennas at the receiver end remains limited in mobile devices owing to their limited size and complexity. Cooperative communication is a possible solution for overcoming this limitation without deploying additional antennas in mobile devices.

In particular, decode-and-forward (DF) protocols are widely adopted in cooperative communications because of their superior performance and signal processing [6,7,8]. However, the DF protocol may offer fewer advantages when the detection error at the relay is properly addressed. To mitigate this error propagation effect, the DF protocol has been widely investigated [9,10,11]. Joint maximum likelihood (JML) detection is a well-known method that can perfectly extract the diversity gain obtained from wireless channels. However, despite its advantages, addressing its complexity at the destination is a challenge. Alternatively, the cooperative maximum ratio combining (MRC) [9] and pseudo-linear combiner [10] methods have been introduced, but their applications are limited to single antennas and orthogonal space-time block codes, respectively. Pairwise error probability (PEP)-based ML detection was introduced in [11] to overcome the above limitations. Nevertheless, the aforementioned studies assume that the source, relay, and destination use an infinite number of analog-to-digital converters (ADCs) at the receiver.

The use of low-resolution analog-to-digital converters (ADCs) is an efficient solution for reducing the power consumption at receivers in future wireless systems, as they exploit a large bandwidth or numerous antennas [12,13,14]. In particular, one-bit ADCs can be used in the future wireless systems by significantly decreasing the power consumption [15,16]. Despite their potential use, only a few studies have investigated one-bit ADCs in cooperative communications [17,18,19,20,21,22,23]. The descriptions of the studies are briefly summarized in Table 1. The performance of multipair massive MIMO cooperative systems were introduced in [17,18] in terms of capacity. In [22,23], a multihop–multi-user MIMO system was investigated, where the non-linearity introduced by one-bit ADCs was overcome through a supervised learning approach. However, this approach involves a significant complexity, making it unfeasible for wireless applications. In particular, the use of a deep learning approach is difficult to implement in mobile devices. Thus, instead of deep learning, a practical detection scheme should be designed with reasonable complexity for one-bit ADCs.

This study considered a detection scheme for cooperative DF MIMO systems using one-bit ADCs. Specifically, this study focused on mitigating the error propagation effect for the system under consideration. To efficiently achieve this outcome, the following contributions are made.

- The PEP of MIMO systems that uses one-bit ADCs is proposed. Because the non-linearity of one-bit ADCs hinders the derivation of an exact PEP, the upper bound of the PEP was analyzed instead. It was shown that the analyzed bound is well upper bounded with the simulated result.
- The low-complexity detection scheme for cooperative DF MIMO systems using one-bit ADCs is proposed. The analyzed bound is easily combined with a PEP-based error propagation model reported in [11] in a weighted distance form. Thus, using this bound, the proposed detection scheme can also be obtained in a single weighted distance form. The advantage offered by this proposed detection is that the low-complexity scheme reported in [24] can also be applied; thus, the computational complexity can be significantly reduced.
- In simulations, the proposed detection method provides a significant gain in one-bit ADCs by mitigating the error propagation effect generated from the relay. In addition, the proposed detection can be applied to arbitrary relay protocols similar to [11].

The remainder of this study is organized as follows. Section 2 introduces a MIMO system using one-bit ADCs. In Section 3, the JML detection and its low-complexity algorithm are described. The DF relay protocol and the proposed detection method are explained in Section 4. The simulation results are presented in Section 5 to verify the effectiveness of the proposed detection method. Finally, the conclusions are presented in Section 6.

### Notation

The superscripts ${\left(\cdot \right)}^{T}$ and ${\left(\cdot \right)}^{H}$ denote the transpose and conjugate transpose matrices, respectively. P(·) denotes the probability of an event. Operators Re(·) and Im(·) denote the real and imaginary parts of the complex number, respectively, while log(·) denotes a logarithmic operation. |·| and ${∥\cdot ∥}_{0}$ denote the absolute value and zero norm (which represents the number of nonzero elements), respectively. An indicator function 𝟙{X} is equal to 1 if event X is true, and 0 if untrue. Sign(x) is a function whose value is 1 for x≥1, and −1 for all other *x*, while $\tilde{x}$ is a function whose value is 1 if x=1, and 0 for all other *x*. An indicator function 𝟙{X} is equal to 1 if event X is true, and 0 if untrue. Sets R and C represent the set of real and complex numbers, respectively.

## 2. System Model

A point-to-point signal model for MIMO systems using one-bit ADCs is described in this section. Using the signal model, a detection rule using a likelihood function is also presented. The proposed signal model and detection rule are used to describe the proposed relay protocol in Section 3 and Section 4, respectively.

### 2.1. Signal Model

**Figure 1:**
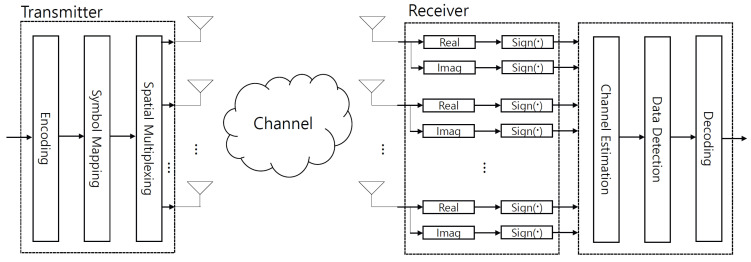
Transmitter and receiver for MIMO systems using one-bit ADCs.

A MIMO system is considered where a node *a* with $N_{a}$ antennas communicate with $N_{b}$ antennas at node *b*. When a signal is transmitted from node *a* over a Rayleigh fading channel, the received signal at node *b* is written as

(1) $$\begin{array}{cc} {\bar{y}}_{b} & =\sqrt{\frac{\gamma_{ab}}{N_{a}}}{\bar{G}}_{ab}{\bar{x}}_{a}+{\bar{w}}_{b}, \end{array}$$

where the average SNR is $\gamma_{ab}$. Channel matrix ${\bar{G}}_{b}\in C^{N_{b}\times N_{a}}$ is Rayleigh fading, whose entries are independent and identically distributed, complex Gaussian random variables with zero mean and unit variance. ${\bar{x}}_{a}\in C^{N_{a}\times 1}$ is the transmitted signal that is modulated from encoded bits (see Figure 1). It belongs to ${\bar{A}}^{N_{a}}$ and is normalized as $\frac{1}{N_{a}}E\{|{\bar{x}}_{a}{|}^{2}\}=1$. $\bar{A}$ is an *M*-quadrature amplitude modulation (QAM) complex constellation set. After receiving the transmitted signal, complex additive white Gaussian noise (AWGN) ${\bar{w}}_{b}\in C^{N_{b}\times 1}$ with zero mean and unit variance is added at the receiver.

To facilitate detection in one-bit ADCs, the complex received signal in (1) is re-expressed as the real signal:

(2) yb=Re{y¯b}Im{y¯b}=γabNaRe{G¯ab}−Im{G¯ab}Im{G¯ab}Re{G¯ab}︸≜GabRe{x¯a}Im{x¯a}︸≜xa+Re{w¯b}Im{w¯b}︸≜wb,=Habxa+wb,

where $H_{ab}≜\sqrt{\frac{\gamma_{ab}}{N_{a}}}G_{ab}\in R^{2N_{b}\times 2N_{a}}$. Signal $x_{a}$ belongs to $A^{2N_{a}}$, where A is a $\sqrt{M}$-pulse-amplitude modulation real-constellation set. The use of one-bit ADCs quantizes the real signal in (2) as

(3) $$\begin{array}{cc} r_{b,i} & =\mathrm{sign}\left(y_{b.i}\right)=\mathrm{sign}\left(h_{ab,i}^{T}x_{a}+w_{b,i}\right), \end{array}$$

where $i\in I=\{1,\ldots ,2N_{b}\}$.

### 2.2. Detection Rule

In this subsection, the ML detection rule is briefly summarized in [24]. After the one-bit ADCs, the receiver first obtains the channel estimates from the quantized signals in (3). Then, data detection is applied to obtain decoded bits using the likelihood function (see Figure 1). When signal $x_{a}$ is transmitted, the likelihood function is calculated as follows:

(4) $$\begin{array}{cc} P\{r_{b}|x_{a}\} & =\prod_{i=1}^{2N_{b}}P\left\{r_{b,i}|c_{ab,i}\left(x_{a}\right)\right\}, \end{array}$$

where $c_{ab,i}\left(x_{a}\right)$ is the *i*-th element of the codeword $c_{ab}\left(x_{a}\right)≜\mathrm{sign}\left(H_{ab}x_{a}\right)$. Note that the codeword $c_{ab}\left(x_{a}\right)\in R^{2N_{b}\times 1}$ can be understood as a noise-free quantized signal. The *i*-th element of the likelihood function in (4) is computed as follows:

(5) $$\begin{array}{cc} P\{r_{b,i}|c_{ab,i}\left(x_{a}\right)\} & =\left\{\begin{array}{cc} p_{ab,i}\left(x_{a}\right) & ,r_{b,i}\neq c_{ab,i}\left(x_{a}\right)\\ 1-p_{ab,i}\left(x_{a}\right) & ,r_{b,i}=c_{ab,i}\left(x_{a}\right), \end{array}\right. \end{array}$$

where the *i*-th element of the likelihood function $p_{ab,i}\left(x_{a}\right)$ is defined as

(6) $$\begin{array}{cc} p_{ab,i}\left(x_{a}\right) & =Q\left(\sqrt{2}\left|h_{ab,i}^{T}x_{a}\right|\right). \end{array}$$

where $Q\left(x\right)=\frac{1}{\sqrt{2\pi }}\int_{x}^{\infty }e^{-u^{2}/2}du$.

By using a log operation, the likelihood function in (4) can be re-expressed as

(7) $$\begin{array}{cc} \log\left(P\{r_{b}|x_{a}\}\right) & =\sum_{i=1}^{2N_{b}}\left[\log\left(p_{ab,i}\left(x_{a}\right)\right)∥r_{b,i}-c_{ab,i}\left(x_{a}\right){∥}_{0}+\log\left(1-p_{ab,i}\left(x_{a}\right)\right)(1-∥r_{b,i}-c_{ab,i}\left(x_{a}\right){∥}_{0})\right]\\ \; & =-\left(\sum_{i=1}^{2N_{b}}\left[w_{ab,i}\left(x_{a}\right)∥r_{b,i}-c_{ab,i}\left(x_{a}\right){∥}_{0}+{\tilde{w}}_{ab,i}\left(x_{a}\right)(1-∥r_{b,i}-c_{ab,i}\left(x_{a}\right){∥}_{0})\right]\right), \end{array}$$

where $w_{ab,i}\left(x_{a}\right)≜-\log\left(p_{ab,i}\left(x_{a}\right)\right)$ and ${\tilde{w}}_{ab,i}\left(x_{a}\right)≜-\log\left(1-p_{ab,i}\left(x_{a}\right)\right)$. The log-likelihood function in (7) is concisely described by introducing the weighted Hamming distance, defined as follows:

(8) $$\begin{array}{cc} d_{w}\left(r_{b},c_{ab}\left(x_{a}\right);w_{ab}\left(x_{a}\right),{\tilde{w}}_{ab}\left(x_{a}\right)\right) & =-\log\left(P\{r_{b}|x_{a}\}\right). \end{array}$$

Using (8), the ML detection rule that maximizes the likelihood function is expressed as:

(9) $$\begin{array}{cc} {\hat{x}}_{a} & =\underset{x_{a}\in A^{2N_{a}}}{\mathrm{argmax}}\log\left(P\{r_{b}|x_{a}\}\right)\\ \; & =\underset{x_{a}\in A^{2N_{a}}}{\mathrm{argmin}}d_{w}\left(r_{b},c_{ab}\left(x_{a}\right);w_{ab}\left(x_{a}\right),{\tilde{w}}_{ab}\left(x_{a}\right)\right). \end{array}$$

Based on (9), the ML detection selects the candidate symbol with the lowest weighted Hamming distance. For the improvement of readability, representative symbols of this study are summarized in Table 2.

## 3. Joint Maximum Likelihood Detection

In this section, a DF relay protocol for MIMO systems using one-bit ADCs is presented. The JML detection rule is then described with the aim of achieving a full diversity gain. However, the implementation of the JML detection rule is considerably complex. As a low-complexity algorithm for JML detection, a PEP-based ML detection rule reported in [11] is also presented.

### 3.1. Joint Maximum Likelihood Detection

**Figure 2:**
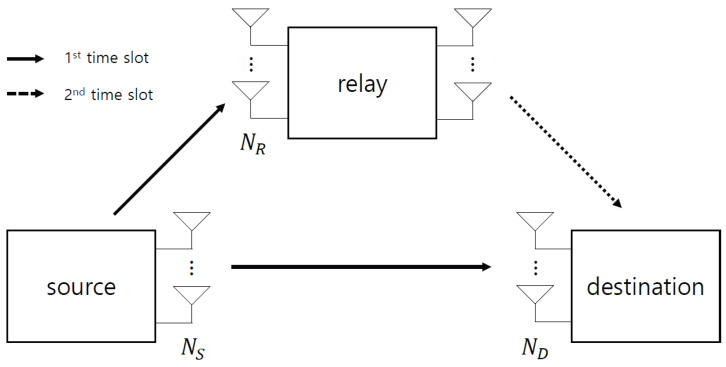
DF relay protocol for the proposed system.

The DF relay protocol is depicted in Figure 2, where the notations *S*, *R*, and *D* denote the source, relay, and destination, respectively. In the first time slot, the source transmits a signal $x_{S}$ to the relay and detection nodes, which is given by:

(10) $$\begin{array}{cc} r_{R} & =\mathrm{sign}(H_{SR}x_{S}+w_{R}),\\ r_{D1} & =\mathrm{sign}(H_{SD}x_{S}+w_{D1}). \end{array}$$

Note that direction communication decodes the signal $x_{S}$ from $r_{D1}$. After receiving this signal $r_{R}$, the relay detects the transmitted signal $x_{S}$ according to the ML criterion in (9), as follows:

(11) $$\begin{array}{cc} x_{R} & =\underset{x_{S}\in A^{2N_{S}}}{\mathrm{argmin}}d_{w}\left(r_{R},c_{SR}\left(x_{S}\right);w_{SR}\left(x_{R}\right),{\tilde{w}}_{SR}\left(x_{S}\right)\right). \end{array}$$

In the second time slot, the relay transmits the detected signal $x_{R}$ to the destination, which is given by:

(12) $$\begin{array}{cc} r_{D2} & =\mathrm{sign}(H_{RD}x_{R}+w_{D2}). \end{array}$$

The destination then detects the transmitted signal $x_{S}$ from quantized signals $r_{D1}$ and $r_{D2}$. The JML criterion [6] is a well-known method for achieving a full diversity gain, which is given by

(13) $$\begin{array}{cc} {\hat{x}}_{S} & =\underset{x_{S}\in A^{2N_{S}}}{\mathrm{argmax}}P\left\{r_{D}|x_{S}\right\}\\ \; & =\underset{x_{S}\in A^{2N_{S}}}{\mathrm{argmax}}P\left\{r_{D1}|x_{S}\right\}\times \sum_{x_{R}\in A^{2N_{S}}}P\left\{x_{R}|x_{S}\right\}P\left\{r_{D2}|x_{R}\right\}, \end{array}$$

where $r_{D}={\left[r_{D1}^{T},\;r_{D2}^{T}\right]}^{T}$. The error probability at the relay associated with a transmitted signal $\left(x_{S}\right)$ is denoted as $P\{x_{R}|x_{S}\}$.

### 3.2. PEP-Based Maximum Likelihood Detection

JML detection in (13) is considerably complex because the total number of candidates to search is given by ${\left|A\right|}^{4N_{S}}$. This cumbersome exercise can be avoided by approximating the error propagation at the relay [11]. In this approximation, the max-log and PEP operations were applied to the JML criterion.

Based on these operations, the likelihood function in (13) for a DF relay protocol is derived as follows:

(14) $$\begin{array}{cc} \log\left(P\{r_{D}|x_{S}\}\right) & =\log\left(P\{r_{D1}|x_{S}\}\right)+\log\left(\sum_{x_{R}\in A^{2N_{S}}}P\left\{x_{R}|x_{S}\right\}P\left\{r_{D2}|x_{R}\right\}\right)\\ \; & \overset{\left(a\right)}{\approx }\log\left(P\{r_{D1}|x_{S}\}\right)+\;\;\;\;\;\;\max_{x_{R}\in A^{2N_{S}}}\log\left(P\left\{x_{R}|x_{S}\right\}P\left\{r_{D2}|x_{R}\right\}\right)\\ \; & \overset{\left(b\right)}{\approx }\log\left(P\{r_{D1}|x_{S}\}\right)+\;\;\;\;\;\;\max_{x_{R}\in A^{2N_{S}}}\log\left(P\left\{x_{S}\to x_{R}\right\}P\left\{r_{D2}|x_{R}\right\}\right), \end{array}$$

where a max-log approximation was used in (a). The conditional error probability is approximated as PEP in (b). Using the likelihood function in (14), the PEP-based ML detection rule [11] is expressed as the sum of the log-likelihood function:

(15) $$\begin{array}{cc} {\hat{x}}_{S} & =\underset{{\left[x_{S}^{T},x_{R}^{T}\right]}^{T}\in A^{4N_{S}}}{\mathrm{argmax}}\log\left(P\{r_{D1}|x_{S}\}\right)+\log\left(P\{x_{S}\to x_{R}\}\right)+\log\left(P\{r_{D2}|x_{R}\}\right), \end{array}$$

where the conditional probabilities of $P\{r_{D1}|x_{S}\}$ and $P\{r_{D2}|x_{R}\}$ are calculated using (4). In addition, $P\{x_{S}\to x_{R}\}$ is the PEP detecting $x_{R}$ when $x_{S}$ is transmitted.

## 4. Proposed Detection

This section presents the proposed detection method that efficiently exploits the PEP-based ML detection of (15) in one-bit ADCs. To efficiently exploit the PEP-based ML detection, the PEP term for (15) of one-bit ADSs should be characterized as a weighted Hamming distance form. To achieve this, the upper bound of the PEP is derived. Subsequently, the proposed detection is derived as a single weighted Hamming distance.

### 4.1. Proposed Detection Rule

#### 4.1.1. Upper Bound of PEP

The log-likelihood functions $\log\left(P\{r_{D1}|x_{S}\}\right)$ and $\log\left(P\{r_{D2}|x_{R}\}\right)$ in (15) can be calculated using (7). However, PEP $\log\left(P\{x_{S}\to x_{R}\}\right)$ is unknown for MIMO systems using one-bit ADCs. The PEP for MIMO systems using infinite-bit ADCs is usually expressed as a *Q* function; however, this expression does not hold for MIMO systems using one-bit ADCs. Moreover, the PEP for the detection rule in (15) should preserve the diversity characteristics of JML detection. Thus, the upper bound of PEP is derived as follows:

**Lemma** **1.** *PEP for MIMO systems using one-bit ADCs between nodes a and b is upper bounded as:*

(16) $$\begin{array}{cc} \log\left(P\{x_{a}\to {\tilde{x}}_{a}\}\right) & \leq -d_{w}\left(c_{ab}\left(x_{a}\right),c_{ab}\left({\tilde{x}}_{a}\right);w_{ab}\left(x_{a},{\tilde{x}}_{a}\right),{\tilde{w}}_{ab}\left(x_{a},{\tilde{x}}_{a}\right)\right), \end{array}$$

*where*$w_{ab,i}\left(x_{a},{\tilde{x}}_{a}\right)$ and ${\tilde{w}}_{ab,i}\left(x_{a},{\tilde{x}}_{a}\right)$ *are, respectively, given by*

$$\begin{array}{cc} w_{ab,i}\left(x_{a},{\tilde{x}}_{a}\right) & =-\log\left(\sqrt{p_{ab,i}\left(x_{a}\right)p_{ab,i}\left({\tilde{x}}_{a}\right)}+\sqrt{\left(1-p_{ab,i}\left(x_{a}\right)\right)\left(1-p_{ab,i}\left({\tilde{x}}_{a}\right)\right)}\right),\\ {\tilde{w}}_{ab,i}\left(x_{a},{\tilde{x}}_{a}\right) & =-\log\left(\sqrt{p_{ab,i}\left(x_{a}\right)\left(1-p_{ab,i}\left({\tilde{x}}_{a}\right)\right)}+\sqrt{\left(1-p_{ab,i}\left(x_{a}\right)\right)p_{ab,i}\left({\tilde{x}}_{a}\right)}\right). \end{array}$$

**Proof.**  For a formal proof derivation, refer to Appendix A. □

#### 4.1.2. Validity of Upper Bound of PEP

**Figure 3:**
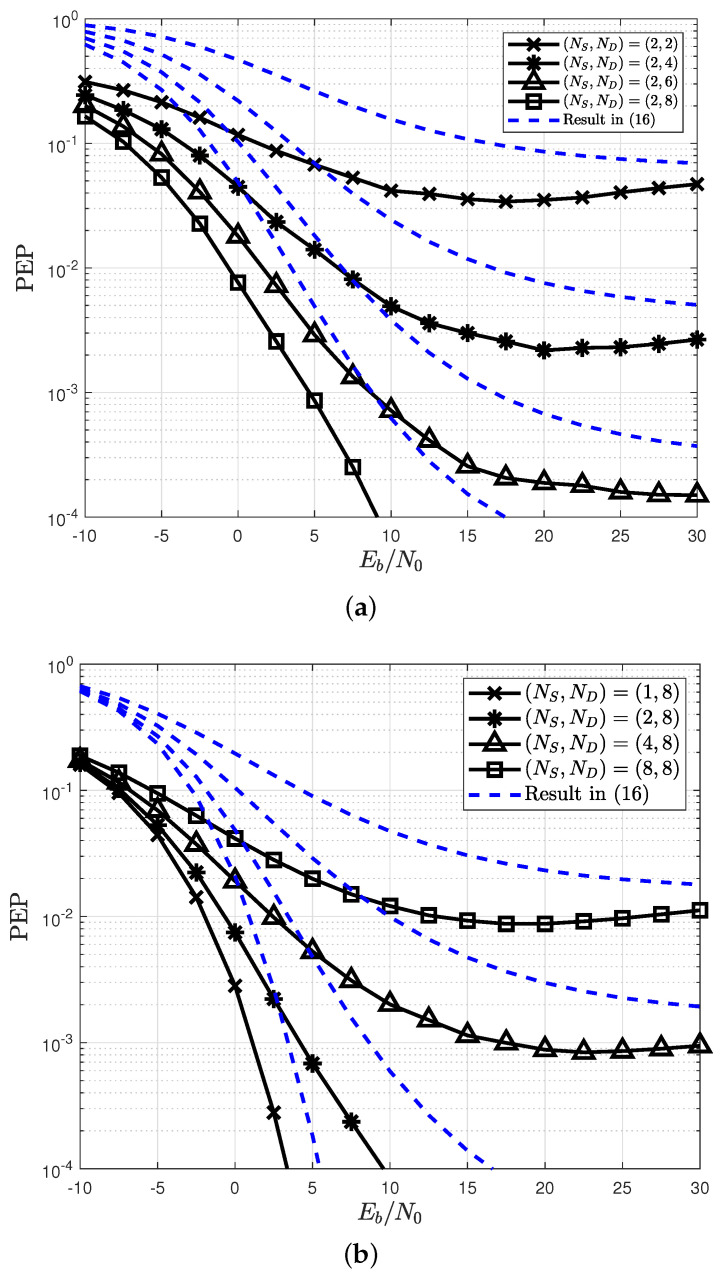
Verification of the weighted Hamming distance for the PEP in Lemma 1. (**a**) Different number of $N_{D}$; (**b**) different number of $N_{S}$.

To demonstrate the validity of Lemma 1, the analysis result in (16) is compared with the simulation result in Figure 3 for different numbers of source and destination antennas. For this simulation, the pairs $\left(x_{a},{\tilde{x}}_{a}\right)$ of $\left(a_{1}^{T},a_{2}^{T}\right)$, $\left({\left[a_{1},a_{1}\right]}^{T},{\left[a_{1},a_{2}\right]}^{T}\right)$, $\left({\left[a_{1},a_{1},a_{1},a_{1}\right]}^{T},{\left[a_{1},a_{1},a_{1},a_{2}\right]}^{T}\right)$, and $\left({\left[a_{1},a_{1},a_{1},a_{1},a_{1},a_{1},a_{1},a_{1}\right]}^{T},{\left[a_{1},a_{1},a_{1},a_{1},a_{1},a_{1},a_{1},a_{2}\right]}^{T}\right)$ were used for $N_{S}=1,2,4,$ and 8, respectively, where $a_{1}=\left[1,1\right]/\sqrt{2}$ and $a_{2}=\left[1,-1\right]/\sqrt{2}$ are the candidate symbols in constellation set A. In Figure 3, the simulated PEP is shown to be upper bound by the analyzed PEP in Lemma 1, regardless of the number of antennas. Note that the PEP is saturated everywhere except at $N_{S}=1$ because it has a saturated performance when $x_{a}\neq -{\tilde{x}}_{a}$ [25].

#### 4.1.3. Proposed Detection

Because the upper bound of PEP is expressed as the weighted Hamming distance between two codewords $c_{ab}\left(x_{a}\right)$ and $c_{ab}\left({\tilde{x}}_{a}\right)$, the detection rule in (15) can be simplified as:

(17) $$\begin{array}{cc} {\hat{x}}_{S}=\underset{{\left[x_{S}^{T},x_{R}^{T}\right]}^{T}\in A^{4N_{S}}}{\mathrm{argmin}} & d_{w}\left(r_{D1},c_{SD}\left(x_{S}\right);w_{SD}\left(x_{S}\right),{\tilde{w}}_{SD}\left(x_{S}\right)\right)\\ \; & +d_{w}\left(r_{D2},c_{RD}\left(x_{R}\right);w_{RD}\left(x_{R}\right),{\tilde{w}}_{RD}\left(x_{R}\right)\right)\\ \; & +d_{w}\left(c_{SR}\left(x_{S}\right),c_{SR}\left(x_{R}\right);w_{SR}\left(x_{S},x_{R}\right),{\tilde{w}}_{SR}\left(x_{S},x_{R}\right)\right). \end{array}$$

Because the detection rule in (17) is represented by the sum of three weighted Hamming distances, the proposed detection rule in (17) can be further summarized as a single weighted Hamming distance:

(18) $$\begin{array}{cc} {\hat{x}}_{S} & =\underset{{\left[x_{S}^{T},x_{R}^{T}\right]}^{T}\in A^{4N_{S}}}{\mathrm{argmin}}d_{w}\left(\left[\begin{array}{c} r_{D1}\\ r_{D2}\\ c_{SR}\left(x_{S}\right) \end{array}\right],\left[\begin{array}{c} c_{SD}\left(x_{S}\right)\\ c_{RD}\left(x_{R}\right)\\ c_{SR}\left(x_{R}\right) \end{array}\right];\left[\begin{array}{c} w_{SD}\left(x_{S}\right)\\ w_{RD}\left(x_{R}\right)\\ w_{SR}\left(x_{S},x_{R}\right) \end{array}\right],\left[\begin{array}{c} {\tilde{w}}_{SD}\left(x_{S}\right)\\ {\tilde{w}}_{RD}\left(x_{R}\right)\\ {\tilde{w}}_{SR}\left(x_{S},x_{R}\right) \end{array}\right]\right). \end{array}$$

From the detection rule in (18), a DF relay protocol with ($N_{S},N_{R},N_{D}$) antennas can be interpreted as a single MIMO system with $\left(N_{S}+N_{R}\right)$ transmitting antennas and $\left(2N_{D}+N_{R}\right)$ receiving antennas. Using this fact, the low-complexity algorithm reported in [24] can be applied to the detection rule reported in (18). Thus, the proposed detection rule can significantly reduce the complexity compared with the JML detection rule. Note that the original PEP-based ML detection reported in [11] also exploits the advantages of an equivalent single MIMO system in the DF relay protocols. However, unlike in [11], the distinct PEP types in (15) are applied to (14). In addition, the proposed detection uses a different low-complexity algorithm reported in [24], whereas the original PEP-based ML detection [11] uses the low-complexity algorithm reported in [26].

### 4.2. Extension to Other Relaying Protocols

**Figure 4:**
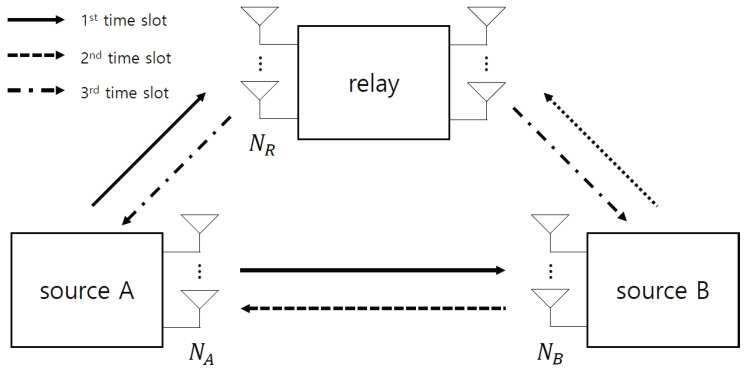
DF protocol for two-way relay.

The proposed detection rule in (18) can be applied to arbitrary space-time codes and relay networks, because the derived PEP can be expressed as a weighted Hamming distance in Lemma 1. For example, the proposed detection can be applied to a two-way relay (TWR) protocol (see Figure 4). Here, source *A* transmits its signal $x_{A}$ to source *B* and relay *R* in the first time slot, which is given by:

(19) $$\begin{array}{cc} r_{B1} & =\mathrm{sign}(H_{AB}x_{A}+w_{B1}),\\ r_{R1} & =\mathrm{sign}(H_{AR}x_{A}+w_{R1}). \end{array}$$

In the second time slot, source *B* transmits its signal $x_{B}$ to source *A* and relay *R*, which is given by:

(20) $$\begin{array}{cc} r_{A2} & =\mathrm{sign}(H_{BA}x_{B}+w_{B2}),\\ r_{R2} & =\mathrm{sign}(H_{BR}x_{B}+w_{R2}). \end{array}$$

After receiving the signals from sources *A* and *B*, the relay detects signals ${\hat{x}}_{A}$ and ${\hat{x}}_{B}$ by using the ML criterion in (9). Then, the relay generates the network-coded signal from the detected signals, which is given by:

(21) $$\begin{array}{cc} x_{R} & =\frac{1}{\sqrt{2}}\left({\hat{x}}_{R,A}+{\hat{x}}_{R,B}\right), \end{array}$$

where complex-field network coding (CFNC) is considered because of its easy implementation while it also guarantees a diversity gain in MIMO systems [27,28]. The relay transmits the network-coded signal from (21) to sources *A* and *B*, which is given by:

(22) $$\begin{array}{cc} r_{A3} & =\mathrm{sign}(H_{RA}x_{R}+w_{A3}),\\ r_{B3} & =\mathrm{sign}(H_{RB}x_{R}+w_{B3}). \end{array}$$

Finally, the signals $r_{A2}$ and $r_{A3}$ are received at source *A*, and the signals $r_{B1}$ and $r_{B3}$ are received at source *B*. Each source performs the proposed detection rule to detect the symbol as:

(23) $$\begin{array}{cc} {\hat{x}}_{A} & =\underset{{\left[x_{A}^{T},x_{R,A}^{T},x_{R,B}^{T}\right]}^{T}\in A^{6N_{S}}}{\mathrm{argmin}}d_{w}\left(\left[\begin{array}{c} r_{B1}\\ r_{B3}\\ c_{AR}\left(x_{A}\right)\\ c_{BR}\left(x_{B}\right) \end{array}\right],\left[\begin{array}{c} c_{AB}\left(x_{A}\right)\\ c_{RB}\left(\frac{x_{R,A}+x_{R,B}}{\sqrt{2}}\right)\\ c_{AR}\left(x_{R,A}\right)\\ c_{BR}\left(x_{R,B}\right) \end{array}\right];\left[\begin{array}{c} w_{AB}\left(x_{A}\right)\\ w_{RB}\left(\frac{x_{R,A}+x_{R,B}}{\sqrt{2}}\right)\\ w_{AR}\left(x_{A},x_{R,A}\right)\\ w_{BR}\left(x_{B},x_{R,B}\right) \end{array}\right],\left[\begin{array}{c} {\tilde{w}}_{AB}\left(x_{A}\right)\\ {\tilde{w}}_{RB}\left(\frac{x_{R,A}+x_{R,B}}{\sqrt{2}}\right)\\ {\tilde{w}}_{AR}\left(x_{A},x_{R,A}\right)\\ {\tilde{w}}_{BR}\left(x_{B},x_{R,B}\right) \end{array}\right]\right). \end{array}$$

(24) $$\begin{array}{cc} {\hat{x}}_{B} & =\underset{{\left[x_{B}^{T},x_{R,A}^{T},x_{R,B}^{T}\right]}^{T}\in A^{6N_{S}}}{\mathrm{argmin}}d_{w}\left(\left[\begin{array}{c} r_{A2}\\ r_{A3}\\ c_{AR}\left(x_{A}\right)\\ c_{BR}\left(x_{B}\right) \end{array}\right],\left[\begin{array}{c} c_{BA}\left(x_{B}\right)\\ c_{RA}\left(\frac{x_{R,A}+x_{R,B}}{\sqrt{2}}\right)\\ c_{AR}\left(x_{R,A}\right)\\ c_{BR}\left(x_{R,B}\right) \end{array}\right];\left[\begin{array}{c} w_{BA}\left(x_{B}\right)\\ w_{RA}\left(\frac{x_{R,A}+x_{R,B}}{\sqrt{2}}\right)\\ w_{AR}\left(x_{A},x_{R,A}\right)\\ w_{BR}\left(x_{B},x_{R,B}\right) \end{array}\right],\left[\begin{array}{c} {\tilde{w}}_{BA}\left(x_{B}\right)\\ {\tilde{w}}_{RA}\left(\frac{x_{R,A}+x_{R,B}}{\sqrt{2}}\right)\\ {\tilde{w}}_{AR}\left(x_{A},x_{R,A}\right)\\ {\tilde{w}}_{BR}\left(x_{B},x_{R,B}\right) \end{array}\right]\right). \end{array}$$

Note that the detection rules in (23) and (24) are, respectively, performed to obtain signals ${\hat{x}}_{A}$ and ${\hat{x}}_{B}$ at the destination at source *B* and source *A*.

### 4.3. Complexity Analysis

In this subsection, the complexity of the proposed detection method is analyzed in terms of real multiplication. In Table 3, the complexity of the JML detection increases exponentially with the number of source antennas. The proposed detection can decrease the complexity by applying the low-complexity algorithm reported in [24], where new design parameters $N_{l}$ and $N_{sv}$ are introduced to reduce the search space dimension. By applying the dimension reduction in [24], the proposed detection is independent of the constellation set; thus, the complexity is significantly reduced by setting the appropriate parameter values of $N_{l}$ and $N_{sv}$. For example, when $\left(N_{S},N_{R},N_{D}\right)=\left(2,8,8\right)$, the number of real multiplication of the JML detection is approximately $3.3\times 10^{7}$, while that of the proposed detection is approximately $2.4\times 10^{6}$.

## 5. Simulations

A coded MIMO system for a DF relay protocol is considered to operate with $\left(N_{S},N_{R},N_{D}\right)=\left(2,8,8\right)$. A frame consists of a pilot and data with 32 and 265 lengths, respectively, and 4-QAM was adopted for symbol mapping. All underlying links were modeled as Rayleigh fading channels, and a linear minimum mean squared error (LMMSE) channel estimation was applied to the pilot. The rate $\frac{1}{2}$ turbo code is adopted for channel coding and is based on parallel concatenated codes with feedforward and feedback polynomials (15,13) in octal notation. In addition, the source-to-relay link was assumed to be obtained at the destination. The low-complexity algorithm [24] with $\left(N_{l},N_{sv}\right)=\left(32,8\right)$ was applied to the proposed detection, where $N_{l}$ and $N_{sv}$ is the list size and dimension of the sub-vector, respectively. As a performance comparison, the MRC and selective decode-and-forward (SDF) methods were simulated using [6,29], respectively. A 16-bit cyclic-redundancy-check scheme was adopted for the SDF where only the corrected signal was forwarded to the destination.

**Figure 5:**
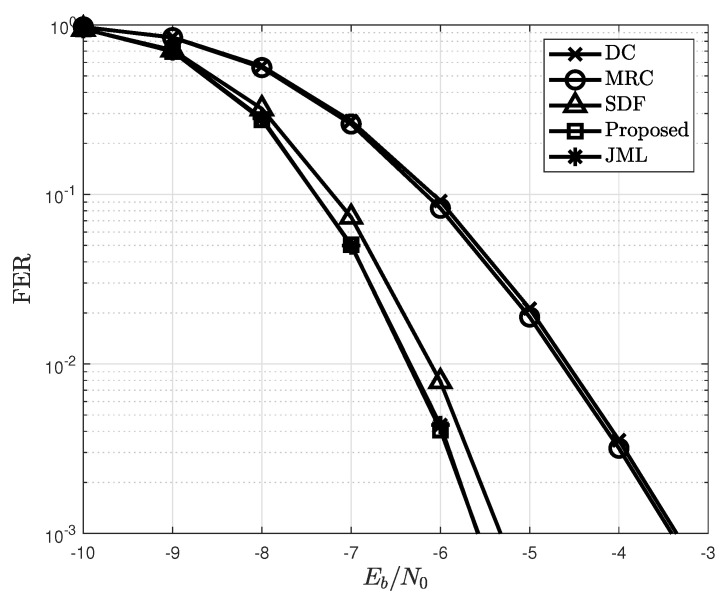
FER of the proposed detection with different detection schemes when $\left(N_{S},N_{R},N_{D}\right)=\left(2,8,8\right)$.

The frame error rate (FER) for different detections is compared with the proposed detection in Figure 5. In this figure, perfect channel state information (PCSI) is assumed at the destination to explicitly check whether the proposed detection achieves a diversity gain. Direct communication (DC) is also simulated in Figure 5 to verify the effectiveness of the relay protocol. The MRC does not achieve a diversity gain from the relay because the detection errors at the relay are not mitigated. In contrast, the proposed detection and SDF efficiently achieve a diversity gain and therefore address the error propagation effect. In particular, the FER of the proposed detection approaches that of the JML with reduced complexity, as shown in Table 3.

**Figure 6:**
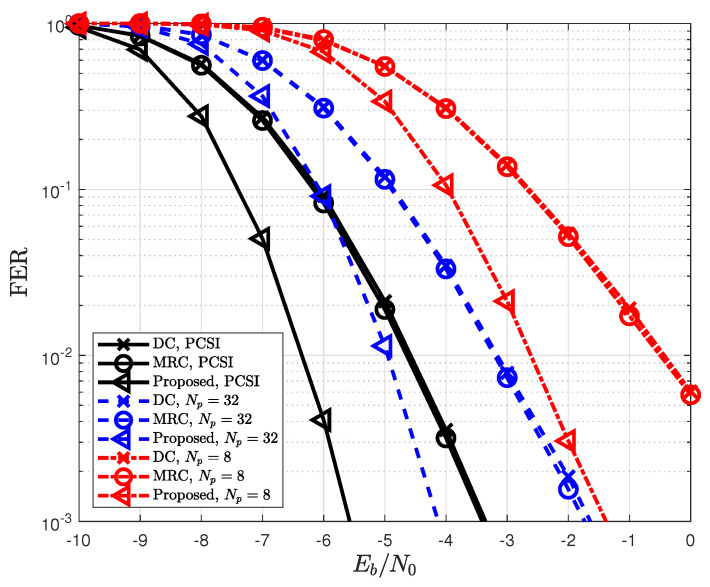
FER for different length of pilots $N_{p}$ when LMMSE channel estimation is applied.

**Figure 7:**
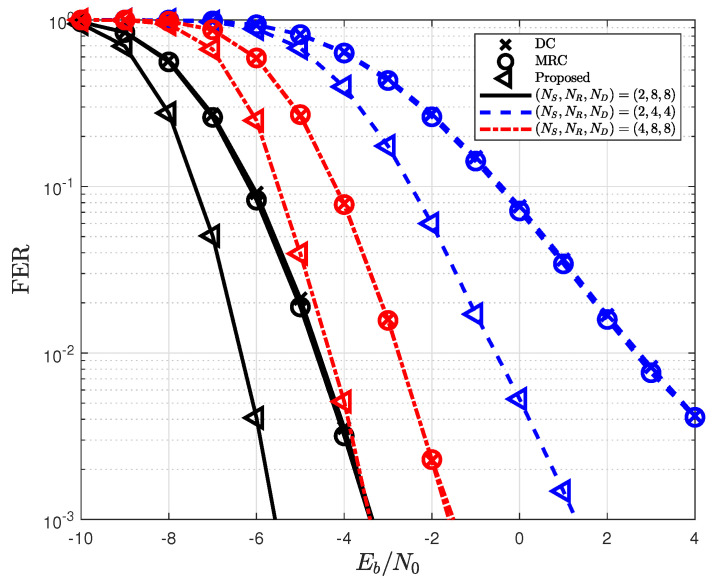
Impact of the number of antenna configurations ($N_{S},N_{R},N_{D}$) on the proposed detection.

In Figure 6, the FER for the proposed detection is depicted according to the number of pilots $N_{p}$. The proposed detection outperforms conventional forms of detection by achieving a diversity gain and is independent of a pilot. However, the FER dramatically decreases as the number of pilots decreases, because the FER of the MIMO system that uses one-bit ADCs is highly dependent on the channel estimation performance. Figure 7 shows the FER based on the number of the source, relay, and destination antennas. As the number of source antennas increases, the FER decreases owing to spatial multiplexing. In contrast, the FER improves with the number of destination antennas because the diversity gain of the proposed detection also increases. From this figure, the antenna configurations of $\left(N_{S},N_{R},N_{D}\right)=\left(2,8,8\right)$ are used in the simulations because a sufficient FER performance cannot be achieved in one-bit ADCs when the number of relay and destination antennas is small.

**Figure 8:**
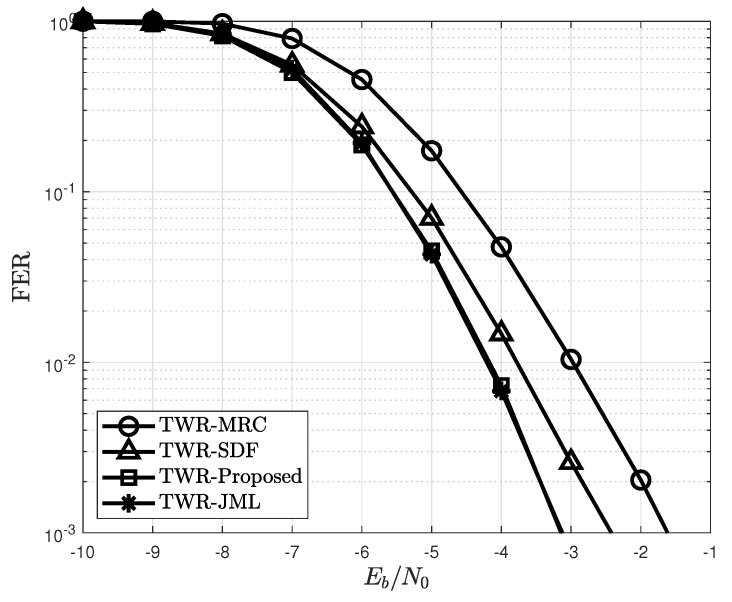
FER of the proposed detection for different detection schemes in a two-way relay.

The FER of the proposed detection in the TWR protocol when $N_{p}=32$ is depicted in Figure 8. Similar to Figure 5, the proposed detection method outperformed conventional detection methods. In particular, the proposed detection achieves an FER similar to that of the JML. This achievement shows that the derived PEP in Lemma 1 is well upper bounded and is applicable to the error propagation model reported in [11]. Thus, the proposed detection method can efficiently mitigate the error propagation effect, regardless of the relay protocols adopted.

## 6. Conclusions

This study considered a detection scheme for cooperative MIMO systems using one-bit ADCs in a DF relay protocol. The detection error at the relay was the main cause of the performance degradation of the DF relay protocol. Especially, the error propagation effect was severe in one-bit ADCs, which produces the significant performance degradation. To efficiently mitigate the error propagation effect caused by the detection error, the upper bound of the PEP was first derived as a weighted Hamming distance. By using the derived PEP, the proposed detection was obtained as a single weighted Hamming distance form, and the low-complexity detection scheme was then applied to this form. The complexity of the proposed detection was analyzed in terms of real multiplications. The simulation results showed that the FER of the proposed detection approaches that of the JML; however, its complexity is considerably reduced.

An interesting direction for future research is to explicitly derive the diversity order for the proposed detection. To achieve this, the analyzed PEP in (16) of the ML detection can be exploited for the PEP calculation of the proposed detection. Then, the diversity order can be derived for the PEP calculation in a similar way to [25]. Other future research is to consider the one-bit transceiver at the nodes. The one-bit transceiver design is challenging research but will significantly increase the system power efficiency.

## Figures and Tables

**Table 1:** Relevant studies for cooperative MIMO systems using one-bit ADCs.

Papers	Descriptions	Performance Measure
[17]	Multipair MIMO relay using one-bit transceivers	Capacity
[18]	Massive MIMO relay using one-bit transceivers	Capacity
[21]	Relay cluster using one-bit transceivers	Error rate
[22]	Multihop relay using one-bit transceivers	Error rate

**Table 2:** Representative symbols in this study.

Symbols ${}^{1}$	Descriptions
$x_{a}$	transmitted signal at node *a*
$H_{ab}$	channel matrix between nodes *a* and *b*
$w_{b}$	noise signal at node *b*
$y_{b}$	received signal at node *b*
$r_{b}$	quantized signal at node *b*
$P\{r_{b}|x_{a}\}$	the likelihood function when signal $x_{a}$ is transmitted
$c_{ab}\left(x_{a}\right)$	(noise-free) codeword between nodes *a* and *b*
$p_{ab,i}\left(x_{a}\right)$	$Q\left(\sqrt{2}\left|h_{ab,i}^{T}x_{a}\right|\right)$
$w_{ab,i}\left(x_{a}\right)$	$-\log\left(p_{ab,i}\left(x_{a}\right)\right)$
$w_{ab,i}\left(x_{a}\right)$	$-\log\left(1-p_{ab,i}\left(x_{a}\right)\right)$
$d_{w}\left(r_{b},c_{ab}\left(x_{a}\right);w_{ab}\left(x_{a}\right),{\tilde{w}}_{ab}\left(x_{a}\right)\right)$	the weighted Hamming distance

^1^ Subscripts *a* and *b* can be either source *(S)*, relay *(R)*, or destination *(D)*.

**Table 3:** Complexity analysis in terms of real multiplications.

Detection Schemes	Number of Real Multiplications	Example ${}^{a}$
JML	$\left(4N_{S}+2\right)N_{D}{\left|A\right|}^{2N_{S}}+\left(\left(4N_{R}+2\right)N_{D}+\left(8N_{S}+12\right)N_{R}\right){\left|A\right|}^{4N_{S}}$	32,526,336
MRC	$\left(\left(4N_{S}+2\right)N_{D}+\left(4N_{R}+2\right)N_{D}\right){\left|A\right|}^{2N_{S}}$	90,112
Proposed detection ${}^{b}$	$\left(\left(4N_{S}+2\right)N_{D}+\left(8N_{S}+12\right)N_{R}+\left(4N_{R}+2\right)N_{D}\right)\frac{4N_{D}^{2}N_{l}^{2}}{N_{sv}^{2}}$	2,359,296 ${}^{c}$

^*a*^ Antennas configuration with *(N_S_, N_R_, N_D_)* = (2, 8, 8) using 4-QAM modulation is applied for this example. ^*b*^ *N_l_* is the list size and *N_sv_* is sub-vector dimension for the low-complexity algorithm in [24]. ^*c*^ Parameters *(N_l_* , *N_sv_)* = (32, 8) are applied for this example.

## Data Availability

Not applicable.

## Appendix A

**Proof** **of** **Lemma A1.** The PEP for detecting ${\tilde{x}}_{a}$ when $x_{a}$ is transmitted is defined as:

(A1) $$\begin{array}{cc} P\{x_{a}\to {\tilde{x}}_{a}\} & =\sum_{r_{b}\in {\left\{-1,1\right\}}^{2N_{b}}}P\left\{r_{b}|x_{a}\right\}𝟙\left\{P\left\{r_{b}|x_{a}\right\}\leq P\left\{r_{b}|{\tilde{x}}_{a}\right\}\right\}, \end{array}$$

where the indicator is upper bounded by $𝟙\left\{A\leq B\right\}\leq {\left(\frac{B}{A}\right)}^{s}$ with s∈[0,1]. By applying $s=\frac{1}{2}$ to (A1), the upper bound of PEP is obtained as:

(A2) $$\begin{array}{cc} P\{x_{a}\to {\tilde{x}}_{a}\} & \leq \sum_{r_{b}\in {\left\{-1,1\right\}}^{2N_{b}}}P\left\{r_{b}|x_{a}\right\}{\left(\frac{P\{r_{b}|{\tilde{x}}_{a}\}}{P\{r_{b}|x_{a}\}}\right)}^{\frac{1}{2}}\\ \; & =\sum_{r_{b}\in {\left\{-1,1\right\}}^{2N_{b}}}\sqrt{P\left\{r_{b}|x_{a}\right\}P\left\{r_{b}|{\tilde{x}}_{a}\right\}}. \end{array}$$

By applying (4) to (A2), the PEP is further computed as:

(A3) $$\begin{array}{cc} P\{x_{a}\to {\tilde{x}}_{a}\} & \leq \sum_{r_{b}\in {\left\{-1,1\right\}}^{2N_{b}}}\prod_{i=1}^{2N_{b}}\sqrt{P\left\{r_{b,i}|c_{ab,i}\left(x_{a}\right)\right\}P\left\{r_{b,i}|c_{ab,i}\left({\tilde{x}}_{a}\right)\right\}}\\ \; & =\prod_{i=1}^{2N_{b}}\underset{≜A}{\underset{︸}{\sum_{r_{b,i}\in \left\{-1,1\right\}}\sqrt{P\left\{r_{b,i}|c_{ab,i}\left(x_{a}\right)\right\}P\left\{r_{b,i}|c_{ab,i}\left({\tilde{x}}_{a}\right)\right\}}}}, \end{array}$$

where term *A* in (A3) can be calculated by dividing two cases: $c_{ab,i}\left(x_{a}\right)=c_{ab,i}\left({\tilde{x}}_{a}\right)$ and $c_{ab,i}\left(x_{a}\right)\neq c_{ab,i}\left({\tilde{x}}_{a}\right)$. For $c_{ab,i}\left(x_{a}\right)=c_{ab,i}\left({\tilde{x}}_{a}\right)$:

(A4) $$\begin{array}{cc} A\; & =\;\sqrt{p_{ab,i}\left(x_{a}\right)p_{ab,i}\left({\tilde{x}}_{a}\right)}\;+\;\sqrt{\left(1-p_{ab,i}\left(x_{a}\right)\right)\left(1-p_{ab,i}\left({\tilde{x}}_{a}\right)\right)}. \end{array}$$

For $c_{ab,i}\left(x_{a}\right)\neq c_{ab,i}\left({\tilde{x}}_{a}\right)$:

(A5) $$\begin{array}{cc} A\; & =\;\sqrt{p_{ab,i}\left(x_{a}\right)\left(1-p_{ab,i}\left({\tilde{x}}_{a}\right)\right)}\;+\;\sqrt{\left(1-p_{ab,i}\left(x_{a}\right)\right)p_{ab,i}\left({\tilde{x}}_{a}\right)}. \end{array}$$

By applying the results of (A4) and (A5) to (A3) and the log operation, the PEP is upper bounded as:

(A6) $$\begin{array}{cc} \log\left(P\{x_{a}\to {\tilde{x}}_{a}\}\right) & \leq -(\sum_{i=1}^{2N_{b}}[w_{ab,i}\left(x_{a},{\tilde{x}}_{a}\right){∥c_{ab,i}\left(x_{a}\right)-c_{ab,i}\left({\tilde{x}}_{a}\right)∥}_{0}\\ \; & +{\tilde{w}}_{ab,i}\left(x_{a},{\tilde{x}}_{a}\right)(1-∥c_{ab,i}\left(x_{a}\right)-c_{ab,i}\left({\tilde{x}}_{a}\right){∥}_{0})]), \end{array}$$

where $w_{ab,i}\left(x_{a},{\tilde{x}}_{a}\right)$ and ${\tilde{w}}_{ab,i}\left(x_{a},{\tilde{x}}_{a}\right)$ are, respectively, defined as

$$\begin{array}{cc} w_{ab,i}\left(x_{a},{\tilde{x}}_{a}\right) & =-\log\left(\sqrt{p_{ab,i}\left(x_{a}\right)p_{ab,i}\left({\tilde{x}}_{a}\right)}+\sqrt{\left(1-p_{ab,i}\left(x_{a}\right)\right)\left(1-p_{ab,i}\left({\tilde{x}}_{a}\right)\right)}\right),\\ {\tilde{w}}_{ab,i}\left(x_{a},{\tilde{x}}_{a}\right) & =-\log\left(\sqrt{p_{ab,i}\left(x_{a}\right)\left(1-p_{ab,i}\left({\tilde{x}}_{a}\right)\right)}+\sqrt{\left(1-p_{ab,i}\left(x_{a}\right)\right)p_{ab,i}\left({\tilde{x}}_{a}\right)}\right). \end{array}$$

(A6) can be transformed into the weighted distance form in (8), which completes the proof. □
